# Examining the effects of a mindfulness-based distance learning professional training module on personal and professional functioning: a qualitative study

**DOI:** 10.1186/s12909-016-0810-2

**Published:** 2016-11-09

**Authors:** Simon Whitesman, Robert Mash

**Affiliations:** 1 Family Medicine and Primary Care, Stellenbosch University, Stellenbosch, South Africa; 2 The Institute for Mindfulness South Africa, Cape Town, South Africa

**Keywords:** Mindfulness, Education, Teaching, Qualitative research, South Africa

## Abstract

**Background:**

Training people to deliver mindfulness-based interventions (MBI) is becoming an important activity as mindfulness has been shown to have clinical benefits across a variety of conditions. Training courses must not only address the principles, skills and theory of mindfulness, but the trainers themselves must be able to embody the practice. There is limited research on the ability of teachers-in-training to embody the practice as a result of teacher training programmes. This study explored the extent to which a short course enabled future teachers to embody mindfulness practice. This first module was part of a larger course of four modules to prepare future teachers of MBIs.

**Methods:**

Qualitative data was obtained from 10 out of 35 end of course written assignments that asked respondents to reflect on their experience of mindfulness practice during the course. These were systematically selected and a focus group interview was also conducted with local participants. Data was analysed by means of the framework method and key themes identified.

**Results:**

The combination of a retreat and on-line learning was perceived to be effective. Students reported significant changes in personal functioning as a result of daily mindfulness practice: self-awareness, improved relationships, enhanced connectedness, better self-regulation, enhanced compassion and curiosity towards self and others and self-acceptance. Participants began to introduce elements of mindfulness into their professional practice.

**Conclusions:**

The first module of a post-graduate training programme for health professionals who want to teach MBIs successfully supported students to embody, explore and apply mindfulness in their lives. The integrated teaching modalities of residential retreat and distance-based on-line learning appeared effective.

## Background

Mindfulness is an inherent human capacity that can be cultivated intentionally through attending to phenomena in the present moment such as our sensations, thoughts or feelings, while holding an attitude of curiosity and non–judgment [1]. Mindfulness has been recognized as a way to enhance self-regulation, emotional intelligence and to foster improved relationships, not only in healthcare, but also in the educational (primary and secondary levels) and corporate sectors [2]. Indeed the UK government have spoken of the need for “a mindful nation” and has promoted practical ways to integrate mindfulness into society and expand access to mindfulness-based programmes in multiple domains such as healthcare, education, the criminal justice system and politics [2]. One of the central requirements for incorporating contemporary approaches to mindfulness into secular society– which is relevant to all countries in which these applications of mindfulness are being offered - is access to mindfulness-based programmes that are taught by competent and well-trained teachers.

The formalisation of mindfulness as a therapeutic clinical intervention has received increasing attention in the last decade as a result of the growing evidence-base for its effectiveness in psychological and social functioning [3]. Medical applications of mindfulness-based programmes, with varying degrees of efficacy, include conditions such as chronic pain, HIV, diabetes, cardiovascular disease, infertility, functional gastrointestinal disorders, malignant disease and infertility. Systematic reviews describe a moderate and consistent positive effect on mental health in somatic conditions, as well as those with mild to moderate psychological issues [3, 4]. No adverse effects were described. There is a paucity of literature describing the impact of standardised mindfulness based programmes on social functioning [3–6].

A number of recommendations have been published to enhance the impact of mindfulness-based interventions (MBI) [7]. With regard to teacher training it was noted that there are”unique expectations for MBI instructors, which require a personal practice in mindfulness meditation in addition to professional training in the clinical approach. This element may challenge future implementation efforts and has received surprisingly little attention to date in the scientific literature.” [7] Various groups are considering how to develop teacher assessment criteria by which training organisations can standardise assessment processes to ensure trainee teachers achieve a minimum level of competence before offering such interventions to the public [8, 9].

Apart from the issue of teacher assessment criteria that mainly address an understanding and ability to disseminate the core practices, ethics and concepts of MBIs, there is a paucity of research investigating the ability of teachers to embody mindfulness. Such research needs to look at their ability to embody mindfulness in terms of enhanced self-awareness, self-compassion and insight, and to explore how this changes over time.

To begin to address this gap, we have begun conducting a series of research projects with trainee teachers undergoing a two-year, four module, professional training in MBIs at Stellenbosch University, South Africa. A previous study demonstrated a measurable improvement in mindfulness, self-compassion and levels of stress as a result of the first module, although no impact on levels of self-determination [10].

The aim of this study was to explore the experience of participants in the first module with regard to learning and adopting mindfulness practice, and any perceived effect on their professional practice or personal functioning.

## Methods

### Study design

This was a qualitative study that made use of data from a focus group interview with course participants and written assignments during the module.

### Setting

The whole training programme consists of four modules structured as a series of short courses of 8–10 weeks in length. The course is offered as a collaboration between Stellenbosch University and the Institute for Mindfulness South Africa. An overview of the whole programme is as follows:

*Module 1: Mindfulness From the Inside-Out: An Introduction to Mindfulness and Mindfulness-Based Approaches.* Core Intent: For participants to experience and explore the practice of mindfulness, (both formally and in daily living), to understand what constitutes MBIs and appreciate the professional context in which these can be applied. Retreat: Mindfulness Practitioner Development retreat; 5-days

*Module 2: Blending Form and Essence: Exploring the Key Elements of a Mindfulness-Based Intervention* Core Intent: For participants to understand the architecture, essential content and process of a MBI by exploring the key elements of Mindfulness-Based Stress Reduction (MBSR) and Mindfulness-Based Cognitive Therapy (MBCT). Retreat: Professional Development Retreat; 5-days

*Module 3: The courage to teach: the person and presence of the teacher.* Core Intent: To explore the centrality of the teacher embodying the essential qualities of mindfulness, while navigating the dynamic space between teacher and participant sensitively and skillfully in an accessible and context-appropriate way. Retreat: Integrating Stillness and Action in the World; Retreat: 7-days

*Module 4:The ground beneath our feet: The Foundations of Mindfulness.* Core Intent: For participants to be exposed to and explore the historical context, and the ethical and compassionate foundation of mindfulness, and its ongoing relevance and applicability in the present day. Retreat: Teacher-led Silent Retreat; 5-days

Methods of learning include residential training retreats and on-line distance learning through discussion forums, written feedback, self-directed mindfulness practice, self-reflective journaling, reading and critiquing peer-reviewed literature and writing essays for assessment purposes.

This study explored the experience of 35 students who were mostly healthcare practitioners and who had completed 9-weeks of study in module 1. Participants were assessed by their contribution to on-line discussions and to two final written assignments, which were submitted at the end of the module. The one assignment was a personal narrative on their experience and exploration of mindfulness practice and the second assignment was a critique of the theory of mindfulness.

The personal narrative was a 1500 word written assignment at the end of the module that explored the following issues:

- An overarching impression of mindfulness practice
- The challenges of cultivating mindful awareness
- The value of mindfulness
- Potential application in their work context
- What they learnt about themselves

### Study population and sampling

All 35 participants registered for the module were asked to give consent for their personal narrative to be used as qualitative data. All participants who lived within the Western Cape were invited to attend a focus group interview.

### Data collection

A focus group interview was conducted by RM in the month after the end of the module. RM was an experienced qualitative researcher and was not involved in teaching on the module. The interview was conducted in English at a neutral convenient venue and lasted about 60 min. An interview guide was used to ensure that the conversation explored all topics of interest. The opening question was “Please tell me about your experience of the module on mindfulness, which you have just completed?” and follow up questions guided discussion on the practice of mindfulness and any effect on personal functioning or professional practice. The interview was audio-recorded. Ten of the written personal narrative assignments were also selected by use of systematic sampling (every third assignment from an alphabetical list of participants).

### Data analysis

A verbatim transcript of the focus group interviews was checked and corrected against the original audio recording. Qualitative data from the written assignments and the transcript was entered into Atlas-ti and analysed using the framework method [11, 12]. This method involved 5-steps:

1. Familiarisation with the data
2. Construction of a thematic index
3. Application of the codes in the index to all qualitative data
4. Charting of the same codes or code families together
5. Interpretation of the charts in terms of the types of themes and range of different opinions/experiences and possible associations between these different themes

BM independently analysed the focus group interview and SW the written assignments. The two researchers then compared and integrated their themes and discussed any difference in their interpretation of the data. The themes are reported using data from all the qualitative data sources.

## Results

Ten assignments were selected for analysis, while 7 students participated in the focus group interview. Out of the 17 participants 16 were women and their profile is further described in Table 1.

**Table 1:** Participant profile

Number	Data Source	Age/years	Professional background
1	Focus group	53	Bereavement counselor
2	Focus group	39	Communication consultant
3	Focus group	46	Director & Trainer PR Company
4	Focus group	50	Dietician
5	Focus group	47	Fitness trainer
6	Focus group	36	General practitioner
7	Focus group	45	Social worker
8	Assignment	55	Facilitator in development sector
9	Assignment	55	Physiotherapist
10	Assignment	69	Counselor
11	Assignment	39	Communication consultant
12	Assignment	61	Nurse practitioner/Health coach
13	Assignment	55	Executive coach
14	Assignment	44	Clinical psychologist
15	Assignment	36	General practitioner
16	Assignment	54	General practitioner
17	Assignment	42	Social Worker

### Experience of the course as a learning experience

Overall the participants were positive about the design of the module as it unfolded week by week. They conceptualised the module as having three important elements: the community of people and their interactions, the practice of mindfulness and the theoretical understanding of what they were experiencing. The course was more intense and demanding than they had anticipated:

*‘I looked at it in terms of the interaction with, with people around you, so you are learning from one another; the opportunity to experience the mindfulness and then the theory because it kind of then cements.’(FGI)*

*“It took more time than I thought it was going to take and somebody said this is like MBSR on steroids.” (FGI)*

The retreat at the start of the module was seen as fundamental to all three elements. The retreat enabled people to bond as a group and engage with each other and the course, which would have been more difficult if they only encountered each other on-line. The retreat also acted as a total immersion in the practice of mindfulness and the modeling of the core values and practice by the facilitators. For some the retreat pushed their boundaries and was transformative. The retreat also included some key theory to start making sense of the practice:

*“I liked the way it started, I think it was very important that it started with the retreat because then the community was formed, the practices were taught and you could kind of really could see were you were going.” (FGI)*

As a whole the group were more orientated towards the practice than the theory of mindfulness and would have liked even more opportunity to practice. Most people however were happy with the process and the balance of practice and theory. Several felt that the theory was important in interpreting and conceptualising their experience through practice. People found some of the more scientific research articles difficult to understand and felt they lacked sufficient scientific literacy. People enjoyed the way that the immersion in practice was followed by an incremental exposure to theory on-line, which helped them make sense of their experience:

*“So I felt like the theoretical underpinnings worked very well with the actual practice. What was being prescribed as a practice, and was really held together by the way the community was pulled together with certain online, … discussion forums or the online discussions.”(FGI)*

*“I wanted to be able to evaluate it, like do I actually agree with what’s [said], you know can I make a critical analysis of this study and I could not do that because I don’t have the post-grad research tools to tackle that.’ (FGI)*

### Effects on mindfulness practice

Respondents appeared to form two groups. The one group had previously developed a mindfulness practice and saw this course as a way of reinforcing, improving or bringing them back to their existing practice. For them the course was a potential part of a longer-term journey with mindfulness. The other group was new to the practice of mindfulness and found the course more transformative.

The experience of the module reinforced the centrality of mindfulness as a daily practice, with participants observing the impact of mindfulness in daily life as a result of participation in the module. Many students recognized that mindful awareness was a fundamentally different mode from the one they were used to (or habituated to) and that this supported a new way of seeing, being and connecting in the world:

“*I’m beginning to understand mindfulness as deep, open connectedness with experiences, with others and with self. Not simply observation ‘of’, but conversation ‘with’, which feels like an entirely different mode.” (Essay 1)*

The practice of becoming aware of the breath within the body was commonly cited as a touch point or anchor to the present moment, and, as such, became a tool to support self-regulation in both normal as well as stressful circumstances:

*“The last nine weeks seem to have also been about the breath that has been the most powerful for me. The simple permission to stop and just breath.” (Essay 2)*

Despite the significant commitment that students made to participate in this module, there was an almost universal recognition of the challenge of cultivating mindfulness on a daily basis, most especially with formal practices such as sitting meditation and body scanning:

*“I would say on the more difficult side is that it has been, I feel deeply resistant to sitting every day, strangely knowing, even knowing what I know, and I have stopped for moments and then I continue.”(FGI)*

“*Carving out time for formal practice, for “doing nothing” has felt like deposing productivity from its throne of unquestioned dominance. It doesn’t step down quietly.”(Essay 3)*

Further, the participants found that they needed to approach this challenge of commitment with increasing levels of self-compassion and kindness in order to implement and sustain their intention:

“*So each day, each week a gentle renewed commitment to do more. With a gentleness not felt before, a carrot rather than a whip.” (Essay 4)*

They reported a range of activities and processes which inhibited, constrained or obstructed the intention to practice daily, from the busyness and demands of daily life to the arising of uncomfortable mental, emotional and physical experiences:

“*In phases, I’ve thrown myself against the walls of my own mind, recoiled at the truth of some of my patterns, and struggled to remain committed to the depth of the practice, when a superficial approach to mindfulness lures with ease and convenience.” (Essay 4)*

However, most of the students recognised this as part of the process and an inimitable element of the practice itself and that even when routine was established it could be easily disrupted by unanticipated changes in other parts of one’s life. However one could re-start and begin again at any time. The willingness to continue to cultivate mindfulness in the face of these challenges usually led to a deepening sense of the value of the practice:

“*Sometimes, it feels natural and deep and clear. Sometimes I’m like a goat tied to a post – fighting to get away. Sometimes I’m there in body, but my mind has gone for a walk, and I’m too lazy to bring it back again and again. But the fact that I’m there at all feels like a good thing.” (Essay 3)*

### Effects on personal functioning

The module appeared to create a significantly different internal stance or perspective towards oneself that led to an external change in personal functioning. Respondents described a range of effects, starting with self-awareness of mind, feelings and body and expanding from this to more attuned relationships and communication and further, to a deepening sense of connectedness to nature and the world that surrounded them:

*“It has brought a balance to my stillness, the sensations in terms of my body and my emotions and my thoughts of, and so it kind of feels like it’s more balanced in that space and so every day I wake up and I feel awake, I feel connected to myself, others and the world around me.’(FGI)*

The range of cognitive effects included enhanced focus and attentional capacity, which allowed more effective self-regulation of mental, emotional and physical states:

*“I spend less time mentally rehearsing how I’m going to get things done and less time consumed with being worried about whether I am going to get everything done. Consequently, I’m more focused and efficient.* “(Essay 5)

*“Bringing focus to the detail of my life has felt like using a magnifying glass not only in the world around me, but in seeing, with greater vividness, the creatures of my internal landscape.” (Essay 6)*

In addition an increase in compassion and curiosity was repeatedly described:

*“Beneath this struggle – possibly because of the struggle - something tender and new and strong and freeing is emerging in me.” (Essay 4)*

*“Previously on being kindly told to take better care of myself, my answer would usually be: “How can I? I have a family and a job and no extra time”. Now it is becoming: How can I not?” (Essay 7)*

*“I found by bringing mindful breath and an attentiveness to these vulnerabilities with a kindness, compassion and curiosity, I would have this sense of reclaiming parts of me and becoming more authentic within myself.” (Essay 3)*

These three elements (attention, curiosity and compassion) constitute the core elements and qualities of mindfulness, and resulted in new ways of seeing the self, others and the world:

*“I have experienced some subtle and profound changes in the way I understand my place in the world, in the way I relate to myself, and the role of mindfulness in my life and vocation.” (Essay 8)*

For many students, the growing insight, attentiveness and compassion has led to a sense of self-acceptance. This is not a neutral state but rather describes a changing relationship with self:

*“So one of the biggest shifts for me seems to be growing self-acceptance with compassion; a deepening of self-acceptance; Its warmer, I sense a loving embrace, before there was a grudging acceptance of my faults, there was less self-judgement and criticism, but I was trying to attain a ‘neutral state’ of judgement versus a warm and caring orientation.” (Essay 8)*

*“I experienced a growing patience with, acceptance of and true interest in myself and the people around me and those in my care.” (Essay 4)*

Participants described increasing flexibility in their range of responses, a disengaging of automatic pilot – a core skill in self-regulation - allowing a witnessing of feelings and assumptions and their limiting nature, even when this was difficult to engage with. This occurred in parallel with an increased awareness of and disidentification from judgmental thoughts. There was an appreciation that, although the benefits experienced were significant, it was not always easy to be mindful:

*“In reperceiving one is called to let go of old patterns and habits…and is liberated to see new ways of responding or coping.” (Essay 9)*

*“When I am asked to reflect on what I am experiencing, my immediate response is usually only a small part of the picture – my initial response is really my assumption about what is happening, my defended knowing, and it is only later that I find the open space for deeper reflection that enables me to see more clearly.” (Essay 4)*

*“I was astounded by the degree to which I could sit in the eye of the emotional turbulence of my young children – and even my stressed spouse – simply noticing my feelings rise and fall.” (Essay 8)*

*“One of the greatest challenges was bringing mindfulness practice into my daily life in respect of moods states that surround some of the activities I engage in during the day.” (Essay 10)*

*“So witnessing and peeling away the layers and layers of judgement has been a daunting and delightful exploration, and a lovely dance between experiential and conceptual knowing.” (Essay 9)*

Mindfulness was described and experienced as a particular mode of being (as opposed to a transient state), which felt inherently connected to self and others:

*“So much of what I thought was important becomes irrelevant. In this mode I don’t need to be good or clever or special. I realise I am ordinary and lovable. And that feels like a good place to start.” (Essay 6)*

The practice invited an increasing recognition of and engagement with the paradoxical nature of reality as seen through present moment awareness. This reflects how students increasingly inhabited the essentially non-dual nature of mindfulness, in which polarities are held and transcended:

*“The closer I come to my felt-sense of myself, the less I am there – or the more I lean into my subjective experience, the greater my sense of objectivity. The closer in I come to the felt-sense of myself, the greater the sense of space and openness. And the more I turn in towards myself, the more I feel connected to my loved ones, strangers and all life on this planet.” (Essay 9)*

### Impact on professional practice

Of those who did describe impact in the professional domain, the reports converged on three elements: introducing the concepts of mindfulness and compassion to patients/clients; introducing and using formal mindfulness practices (such as the body scan and awareness of breathing meditation) in therapeutic encounters; and the impact on one’s effectiveness as a clinician or coach. The commonest techniques used involved breathing exercises to reduce stress and becoming more aware of tension or feelings in one’s body:

*“The sort of sense of stress, you could see it in some of the bodies that come in and they [peer counsellors] would be telling a story and the shallow breathing and all of that so we would always just stop, if it was something really drastic, we would stop, we would light a candle for whoever they were talking about and then we would get them to breath, so they would get that, so … we took it back to the body and we took it back down to the breath so that they could also manage it differently.’(FGI)*

A few people had not found any way of employing the techniques in their professional practice while others had not explicitly labeled what they were doing as mindfulness, and only recognized that was what they were doing on reflection.

As has been reported elsewhere in this analysis, the enhancement of self-compassion and compassion towards others, was an emerging thread in personal functioning, which was also reported on in professional contexts:

*“I have begun introducing mindful practices into my counseling, particularly ideas of self-compassion. I find it a powerful way to assist clients [to] feel good about themselves and be more resilient in coping with their pain and suffering.” (Essay 6)*

Significantly, especially amongst those in a clinical context (medicine, mental health), the enhanced self-awareness arising out of personal mindfulness practice positively impacted the therapeutic encounter, enhancing the quality of relatedness, in particular:

“*I experienced growing patience with, acceptance of and true interest in myself and the people around me and those in my care.” (Essay 4)*

Other clinical skills that were improved included listening, effective responding and heightening curiosity, rather than judgment:

*“I have found that my mindful practice also helps me stay more focused and present with my clients. There is a greater awareness of client’s body language and what remains unsaid.” (Essay 6)*

“*I have a greater capacity in the first place to really listen to my patients…to pick up the unspoken, to be able to evaluate what the real need is.” (Essay 7)*

In parallel to this, there were reports of more effective self-regulation during therapeutic and clinical encounters within the clinicians:

“*I am becoming more discerning and working smarter with my time and energy.” (Essay 4)*

## Discussion

Students described a broad range of impacts on self-perception and engagement in the world that moved them in the direction of enhanced self-awareness, −regulation and -compassion. The module appeared to achieve its goal of introducing and exploring personal mindfulness practice. These qualitative findings supported the quantitative results of the previous study that also measured increased mindfulness and self-compassion amongst those completing the first module of the course [10]. The more limited effect on professional practice was not surprising given that the first module is focused on developing a strong foundation in personal practice while modules 2, 3 and 4 focus in greater depth on professional application.

Notwithstanding that certain elements of the module could be improved these data suggest that the structure and process of both retreat and distance learning were mutually reinforcing teaching modalities that were effective at generating a shift in the internal stance of the teachers-in-training of mindfulness-based approaches.

Our initial quantitative data suggested that the first module of this professional training was effective in increasing mindfulness as well as its key attitudinal dimension (viz. kindness, self-acceptance and compassion) [9]. The qualitative analysis presented in this paper offer some insight into how these changes might occur. The narratives reflect that developing a flexible attentional capacity using the body as foundation begins to generate a changing relationship with the thinking mind, including the habitual tendency towards self-judgment. This quality of refined somatic awareness and de-centering from thoughts creates a shift in perspective, which appears to naturally (and often effortlessly) lead to enhanced self-compassion, and by extension, compassion towards others. Subsequent research will examine whether these qualities that are developed through personal practice by teachers-in-training are maintained in a teaching environment and effectively communicated both explicitly and implicitly when delivering mindfulness-based programmes to the public.

While training programmes differ in structure and content, there is increasing standardisation of core themes and assessment criteria in the pedagogy of mindfulness [10]. The particular form of the training programme at Stellenbosch University places strong emphasis on incorporating retreats into each module (without lessening the importance of the distance learning elements). These follow classic retreat structure in the Buddhist lineage (such as early morning practices, extended periods of silence) in addition to engagement with pedagogical elements. The focus group narrative in this qualitative analysis reflects that in the case of the first module, there were many benefits to this with respect to group cohesion, a safe and invitational space to explore different dimensions of practice and ‘sensing’ the embodied qualities of the retreat leaders.

There is a consistent and significant emphasis internationally that mindfulness teachers should embody the core qualities of the practice [8, 9, 13]. As such, consistent shifts in embodied awareness of those undergoing training to offer programmes to the public is a minimum requirement to teach MBIs, with the recognition that this inner transformation in and of itself does not guarantee becoming an effective teacher, but is a prerequisite for becoming one nonetheless. The foundation level (viz. module 1) for this particular approach to MBI teacher development is congruent with this essential ingredient of training.

The value of developing and training health professionals who can both embody and impart mindfulness-based skills in the clinical context has the potential to affect patient care in a number of ways. This is a particularly pressing issue in South Africa given the harsh, stressful and often traumatic conditions in the healthcare environment, especially in public sector facilities [14, 15]. Intensive training in mindfulness enhances self-regulation, resilience and compassion, with the consequent enhanced capacity to manage stress and reduce burnout [16]. These capacities should also enhance patient-centeredness and patient-care, both of which are diminished in the face of excessively stressful clinical demands. In addition, clinician-educators who are empathic, compassionate and effectively self-regulate in the face of demands may impart these qualities to their students (either under- or post- graduates) either implicitly through embodiment, or explicitly through the teaching of mindfulness-based skills in clinical settings.

One of the questions that arise is what are the specific factors within the training programme that effectively generate sustainable inner transformation within the student? For example, do these factors reside entirely within the formal practices, the community experience, the theoretical learning or engagement with experienced teacher-trainers or some combination of these elements?

### Limitations

Not all people were included in the focus group interview and not all essays were analysed. It is possible, therefore, that different views or perceptions might have been obtained if they had all been selected. There was however substantial congruency in the triangulation of views elicited in the focus group interview and essays as well as with the quantitative findings [10]. The group of participants was highly self-selected, as would be the case for any training of this nature, and the findings might have been different with a less motivated group.

### Implications and recommendations

It may be important to consider whether the internal personal changes reported here are maintained on completion of the training programme and beyond, and if so how they are maintained, and if not, what prevents these changes from becoming embedded.

These data describe both benefits as well as challenges of practicing mindfulness and reflect the commonly used teaching maxim that cultivating mindful awareness is simple, but not easy. Further research should investigate the factors that support a learning environment and training process that engages optimally with the difficulties while retaining connection with the inherent simplicity of the practice.

It will also be useful to evaluate how participant’s practice of mindfulness and teaching of MBIs evolve over the whole 2-year course and all four modules.

## Conclusion

The first module of a post-graduate training programme for health professionals who want to teach MBIs successfully supported students to embody, explore and apply mindfuness in their lives. The integrated teaching modalities of residential retreat and distance-based on-line learning, while challenging, were well-received, and appeared effective at enabling participants to learn and adopt mindfulness practice. Application of learning to participant’s professional contexts was limited and subsequent modules focus on this in more depth and as such, a greater impact on professional functioning is to be expected later in the training.

## Abbreviations

FGI: Focus group interview
HIV: Human immunnodeficiency virus
MBCT: Mindfulness-based cognitive therapy
MBI: Mindfulness-based interventions
MBSR: Mindfulness-based stress reduction
UK: United Kingdom
